# Geographic Distribution of Racial Differences in Prostate Cancer Mortality

**DOI:** 10.1001/jamanetworkopen.2020.1839

**Published:** 2020-03-31

**Authors:** Sean A. Fletcher, Maya Marchese, Alexander P. Cole, Brandon A. Mahal, David F. Friedlander, Marieke Krimphove, Kerry L. Kilbridge, Stuart R. Lipsitz, Paul L. Nguyen, Toni K. Choueiri, Adam S. Kibel, Quoc-Dien Trinh

**Affiliations:** 1 Division of Urological Surgery, Harvard Medical School, Brigham and Women’s Hospital, Boston, Massachusetts; 2 Center for Surgery and Public Health, Harvard Medical School, Brigham and Women’s Hospital, Boston, Massachusetts; 3 James Buchanan Brady Urological Institute and Department of Urology, The Johns Hopkins University School of Medicine, Baltimore, Maryland; 4 Department of Radiation Oncology, Harvard Medical School, Dana-Farber Cancer Institute/Brigham and Women’s Hospital, Boston, Massachusetts; 5 Lank Center for Genitourinary Oncology, Harvard Medical School, Dana-Farber Cancer Institute, Boston, Massachusetts

## Abstract

**Importance:**

While racial disparities in prostate cancer mortality are well documented, it is not well known how these disparities vary geographically within the US.

**Objective:**

To characterize geographic variation in prostate cancer–specific mortality differences between black and white men.

**Design, Setting, and Participants:**

This cohort study included data from 17 geographic registries within the Surveillance, Epidemiology, and End Results (SEER) database from January 1, 2007, to December 31, 2014. Inclusion criteria were men 18 years and older with biopsy-confirmed prostate cancer. Men missing data on key variables (ie, cancer stage, Gleason grade group, prostate-specific antigen level, and survival follow-up data) were excluded. Analysis was performed from September 5 to December 25, 2018.

**Exposure:**

Patient SEER-designated race (ie, black, white, or other).

**Main Outcomes and Measures:**

Fine and Gray competing-risks regression analyses were used to evaluate the difference in prostate-cancer specific mortality between black and white men. A stratified analysis by Gleason grade group was performed stratified as grade group 1 and grade groups 2 through 5.

**Results:**

The final cohort consisted of 229 771 men, including 178 204 white men (77.6%), 35 006 black men (15.2%), and 16 561 men of other or unknown race (7.2%). Mean (SD) age at diagnosis was 64.9 (8.8) years. There were 4773 prostate cancer deaths among white men and 1250 prostate cancer deaths among black men. Compared with white men, black men had a higher risk of mortality overall (adjusted hazard ratio [AHR], 1.39 [95% CI, 1.30-1.48]). In the stratified analysis, there were 4 registries in which black men had worse prostate cancer-specific survival in both Gleason grade group 1 (Atlanta, Georgia: AHR, 5.49 [95% CI, 2.03-14.87]; Greater Georgia: AHR, 1.88 [95% CI, 1.10-3.22]; Louisiana: AHR, 1.80 [95% CI, 1.06-3.07]; New Jersey: AHR, 2.60 [95% CI, 1.53-4.40]) and Gleason grade groups 2 through 5 (Atlanta: AHR, 1.88 [95% CI, 1.46-2.45]; Greater Georgia: AHR, 1.29 [95% CI, 1.07-1.56]; Louisiana: AHR, 1.28 [95% CI, 1.07-1.54]; New Jersey: AHR, 1.52 [95% CI, 1.24-1.87]), although the magnitude of survival difference was lower than for Gleason grade group 1 in each of these registries. The greatest race-based survival difference for men with Gleason grade group 1 disease was in the Atlanta registry.

**Conclusions and Relevance:**

These findings suggest that population-level differences in prostate cancer survival among black and white men were associated with a small set of geographic areas and with low-risk prostate cancer. Targeted interventions in these areas may help to mitigate prostate cancer care disparities at the national level.

## Key Points

**Question:**

How do race-based disparities in prostate cancer outcomes differ geographically within the US?

**Findings:**

In this cohort study of 229 771 men in 17 geographic registries within the Surveillance, Epidemiology, and End Results database, black men had a higher risk of mortality overall compared with white men. The greatest race-based survival difference was seen in men with low-risk prostate cancer in the Atlanta, Georgia, registry, where mortality risk among black men was increased more than 5-fold.

**Meaning:**

These findings suggest that race-based survival differences in prostate cancer vary regionally, which may allow for targeted interventions to mitigate these disparities.

## Teaser

This cohort study examines differences in prostate cancer mortality in white vs black men stratified by geographic region.

## Introduction

Black men are more likely than white men to be diagnosed with and die of prostate cancer.^1^ Current evidence attributes this to racial differences in both tumor biology and access to care.^2,3,4^ These differences may also be greatest in specific disease states, such as low-risk prostate cancer.^5,6^

While race-based prostate cancer survival differences have been identified in population-based samples,^7^ little is known about how these differences vary geographically within the US. Because populations of racial and ethnic minority groups are concentrated in certain geographic areas of the US, it is possible that geographic differences in cancer survival may yield apparent race-based differences in survival and vice versa. Indeed, the mortality for many conditions, ranging from cardiovascular disease to cancer, varies significantly among counties. While it is possible that the underlying geographic variation may be associated with biology and clusters of biologically related individuals, it is also plausible that such variation is associated with other factors, such as access to insurance and high-quality care.

In a 2018 study,^2^ we found that there were significant differences in prostate cancer care among hospitals, wherein nearly half the examined hospitals were more likely to provide definitive therapy to white men compared with black men. Moreover, we found that hospitals that primarily treated people in racial minority groups were associated with lower odds of definitive therapy and longer time to definitive therapy, regardless of patient race.^2,4^ Additionally, we found that when patient and sociodemographic factors are taken into account, the differences were actually reversed, that is, black men may have had better survival than white men.^3^ Taken together, these findings may provide more evidence of a racial disparity, suggesting a form of social injustice rather than racial difference attributable to biology.

To better understand the extent to which geographic variability may underlie observed differences in survival associated with race, we used a nationally representative cancer database to characterize geographic registry-based variation in prostate cancer–specific mortality differences between black and white men. We hypothesized that differences in prostate cancer outcomes associated with race would be greatest in areas with large populations of racial minority groups and significant social and economic barriers to health care.

## Methods

### Data Source

This cohort study used the Surveillance, Epidemiology, and End Results (SEER) database, a collection of 18 defined geographic areas (ie, registries) sponsored by the US National Cancer Institute. These registries include Alaska Native Tumor Registry; Connecticut; Detroit (Metropolitan), Michigan; Atlanta (Metropolitan), Georgia; Greater Georgia; Rural Georgia; San Francisco-Oakland metropolitan statistical area, California; San Jose-Monterey, California; Greater California; Hawaii; Iowa; Kentucky; Los Angeles, California; Louisiana, New Mexico, New Jersey, Seattle (Puget Sound), Washington; and Utah. The database covers about one-third of the US population, and the registries are chosen to be nationally representative.^8^ The SEER database contains detailed data on patient sociodemographic and tumor characteristics as well as survival and treatment patterns. The reporting of our methods are consistent with Strengthening the Reporting of Observational Studies in Epidemiology (STROBE) reporting guideline. The Brigham and Women’s Hospital institutional review board deemed this study exempt from review and informed consent because data were deidentified.

### Cohort Selection

We identified men 18 years and older with biopsy-confirmed prostate cancer, including all stages and grades, between January 1, 2007, and December 31, 2014. We chose this time frame because 2007 was the first year that the full complement of covariates was available, including insurance status, and 2014 was the last year of available follow-up data at the time of analysis. We excluded men who were missing information on cancer stage, grade, prostate-specific antigen level, and survival follow-up data. We also excluded men in the Alaska Native Tumor Registry, as there are neither black nor white individuals with prostate cancer collected in this registry.

### Outcomes

The outcome of interest was prostate cancer–specific mortality, based on the SEER variable for cause-specific death classification. We investigated mortality differences between races in the entire cohort. Given that a recent study showed important differences in mortality between black and white men with low-risk prostate cancer,^5^ we also performed the analysis stratified by Gleason grade group, as grade group 1 (low risk) vs grade groups 2 through 5 (intermediate to high risk).^9,10^

### Exposure Variable

The exposure of interest was self-reported patient race, classified as black, white, or other. We fit an interaction term between race and registry to test whether the association of race with the study outcome was statistically significantly different across 18 component registries that compose SEER.

### Covariates

Covariates included demographic characteristics, such as age, county-level median household income, county-level education level (based on percentage of adults without a high school degree), year of diagnosis, and insurance status (insured vs uninsured). We also included clinical characteristics, such as prostate-specific antigen value, Gleason grade group, clinical TNM stage, and receipt of definitive treatment (ie, radical prostatectomy or any form of radiation therapy).

### Statistical Analysis

We reported descriptive statistics using frequencies and proportions for categorical variables; medians and interquartile ranges were used for continuous variables. For baseline patient characteristics, we used the χ^2^ test for categorical variables and *t* test for continuous variables. We constructed Fine and Gray competing risks regression models, including an unadjusted model and an adjusted model with an interaction term between race and registry to assess the association of race with cancer-specific mortality in each registry. Death due to prostate cancer was the primary event of interest, and death due to any other cause was the competing event. We ranked registries by the estimated adjusted hazard ratio (AHR) of prostate cancer–specific mortality for black vs white men. We defined 2-sided statistical significance as *P* < .05. Analyses were performed using Stata statistical software version 14.0 (StataCorp) and R statistical software version 3.4.1 (R Project for Statistical Computing). Analysis was performed from September 5 to December 25, 2018.

## Results

A total of 406 628 men fit our initial inclusion criteria. We excluded 176 762 men (43.5%) for missing data and 95 men (<0.1%) from the Alaska Native cancer registry because this registry contained no black or white men. Our final study sample included 229 771 men (mean [SD] age at diagnosis, 64.9 [8.8] years), of whom 35 006 (15.2%) were black and 178 204 (77.6%) were white. Median (interquartile range) follow-up time was 56 (31-78) months for white men and 53 (27-75) months for black men. Mean (SD) age at diagnosis was 65.2 (8.7) years for white men and 62.6 (8.8) years for black men. Black men were more likely than men of other races to be uninsured (black: 1057 men [3.0%]; white: 2059 men [1.2%]; other: 204 men [1.2%]), have low education level (black: 21 731 men [62.1%]; white: 83 655 men [47.0%]; other: 7261 men [43.9%]), and have low income (black: 21 318 men [60.9%]; white: 85 582 men [48.0%]; other: 6047 men [36.5%]) (Table 1). There were 4773 prostate cancer deaths among white men (2.7%) and 1250 prostate cancer deaths among black men (3.6%). Receipt of definitive treatment (ie, prostatectomy or radiation treatment) was recorded for 125 493 white men (70.4%) and 23 699 black men (67.7%). In the competing-risks regression, black men had the highest risk of mortality (AHR, 1.39; 95% CI, 1.30-1.48). Table 2 details the population of patients by race within each registry. The eTable in the Supplement presents additional characteristics of each registry.

**Table 1.** Descriptive Characteristics of Men With Prostate Cancer in the Surveillance, Epidemiology, and End Results Database From 2007 to 2014

Characteristic	Men, No. (%)				*P* value
	Total (N = 229 771)	White (n = 178 204)	Black (n = 35 006)	Other or unknown (n = 16 561)	
Age at diagnosis, y					
<50	8267 (3.6)	5559 (3.1)	2278 (6.5)	430 (2.6)	<.001
50-59	55 571 (24.2)	41 460 (23.3)	10 692 (30.5)	3419 (20.6)	
60-69	98 880 (43.0)	77 507 (43.5)	14 484 (41.4)	6889 (41.6)	
70-79	55 234 (24.0)	44 143 (24.8)	6465 (18.5)	4626 (27.9)	
≥80	11 819 (5.1)	9535 (5.4)	1087 (3.1)	1197 (7.2)	
Income^a^					
Low	112 947 (49.2)	85 582 (48.0)	21 318 (60.9)	6047 (36.5)	<.001
High	116 773 (50.8)	92 575 (52.0)	13 686 (39.1)	10 512 (63.5)	
Education level^b^					
Low	112 647 (49.0)	83 655 (47.0)	21 731 (62.1)	7261 (43.9)	<.001
High	117 073 (51.0)	94 502 (53.0)	13 273 (37.9)	9298 (56.2)	
Insurance status					
Insured	210 456 (91.6)	164 307 (92.2)	31 721 (90.6)	14 428 (87.1)	<.001
Uninsured	3320 (1.4)	2059 (1.2)	1057 (3.0)	204 (1.2)	
Unknown	15 995 (7.0)	11 838 (6.6)	2228 (6.4)	1929 (11.7)	
Gleason grade group					
1	52 452 (22.8)	41 802 (23.5)	7368 (21.1)	3282 (19.8)	<.001
2	36 569 (15.9)	28 596 (16.1)	5704 (16.3)	2269 (13.7)	
3	13 823 (6.0)	10 708 (6.0)	2136 (6.1)	979 (5.9)	
4	95 934 (41.8)	73 755 (41.4)	14 596 (41.7)	7583 (45.8)	
5	30 993 (13.5)	23 343 (13.1)	5202 (14.9)	2448 (14.8)	
Receipt of definitive treatment^c^					
No	70 691 (30.8)	52 711 (29.6)	11 307 (32.3)	6673 (40.3)	<.001
Yes	159 080 (69.2)	125 493 (70.4)	23 699 (67.7)	9888 (59.7)	

^a^ High income was defined as income above the 50th percentile of county-level median household income, and low income was defined as income at or below the 50th percentile of county-level median household income.

^b^ Low education was defined as individuals with less than a high school diploma or equivalent, and high education included individuals with a high school diploma or equivalent or higher.

^c^ Defined as prostatectomy or radiation treatment.

**Table 2.** Population of Men With Prostate Cancer by Race Within Each Registry of the Surveillance, Epidemiology, and End Results Database From 2007-2014

Registry	Men, No. (%)			
	White (n = 178 204)	Black (n = 35 006)	Other or unknown (n = 16 561)	Total (N = 229 771)
Connecticut	8348 (4.7)	1047 (3.0)	353 (2.1)	9748 (4.2)
Detroit (Metropolitan), Michigan	8580 (4.8)	3609 (10.3)	452 (2.7)	12 641 (5.5)
Atlanta (Metropolitan), Georgia	4992 (2.8)	4029 (11.5)	229 (1.4)	9250 (4.0)
Greater Georgia	12 203 (6.9)	5369 (15.3)	162 (1.0)	17 734 (7.7)
Rural Georgia	305 (0.2)	201 (0.6)	3 (<0.1)	509 (0.2)
San Francisco-Oakland metropolitan statistical area, California	9393 (5.3)	1797 (5.1)	2336 (14.1)	13 526 (5.9)
San Jose-Monterey, California	5840 (3.3)	317 (0.9)	1250 (7.6)	7407 (3.2)
Greater California	41 709 (23.4)	3589 (10.3)	4414 (26.7)	49 712 (21.6)
Hawaii	894 (0.5)	84 (0.2)	1883 (11.4)	2861 (1.3)
Iowa	8207 (4.6)	188 (0.5)	117 (0.7)	8512 (3.7)
Kentucky	9661 (5.4)	900 (2.6)	151 (0.9)	10 712 (4.6)
Los Angeles, California	13 230 (7.4)	3474 (9.9)	2511 (15.2)	19 215 (8.4)
Louisiana	10 287 (5.8)	5318 (15.2)	129 (0.8)	15 734 (6.9)
New Mexico	4183 (2.4)	115 (0.3)	248 (1.5)	4546 (2.0)
New Jersey	22 344 (12.5)	4116 (11.8)	1414 (8.5)	27 874 (12.1)
Seattle (Puget Sound), Washington	11 986 (6.7)	792 (2.3)	777 (4.7)	13 555 (5.9)
Utah	6042 (3.4)	61 (0.2)	132 (0.8)	6235 (2.7)

The stratified multivariable analyses of individuals with Gleason grade group 1 disease and individuals with Gleason grade groups 2 through 5 disease revealed that the prostate cancer–specific mortality difference between black and white men was present in both groups but with a larger effect size for Gleason grade group 1 disease. Within this group, statistically significantly worse survival for black men was seen in 4 of the 17 included registries, and the greatest race-based survival difference for those with Gleason grade group 1 disease was in the Atlanta registry (AHR, 5.49; 95% CI, 2.03-14.87), followed by New Jersey (AHR, 2.60; 95% CI, 1.53-4.40), Greater Georgia (AHR, 1.88; 95% CI, 1.10-3.22), and Louisiana (AHR, 1.80; 95% CI, 1.06-3.07). There were no race-based survival differences for men with Gleason grade group 1 disease in the other registries. For men with Gleason grade groups 2 through 5 disease, survival differences were seen in Detroit (AHR, 1.65; 95% CI, 1.32-2.08), Atlanta (AHR, 1.88; 95% CI, 1.46-2.45), New Jersey (AHR, 1.52; 95% CI, 1.24-1.87), Greater Georgia (AHR, 1.29; 95% CI, 1.07-1.56), and Louisiana (AHR, 1.28; 95% CI, 1.07-1.54); however, the effect sizes were smaller than that seen in Gleason grade group 1 disease for all registries besides Detroit (Table 3).

**Table 3.** Adjusted Hazard Ratio Analysis With Gleason Grade 1 and Grades 2 Through 5 Disease for Prostate Cancer–Specific Mortality Among Black Men Stratified by Region

Registry	AHR (95% CI)^a^	*P* value
Gleason grade group 1		
Connecticut	1.58 (0.47-5.39)	.46
Detroit (Metropolitan)	1.61 (0.52-4.92)	.41
Atlanta (Metropolitan)	5.49 (2.03-14.87)	.001
Greater Georgia	1.88 (1.10-3.22)	.02
Rural Georgia^b^	NA	NA
San Francisco-Oakland metropolitan statistical area, California	0.94 (0.27-3.20)	.93
San Jose-Monterey, California^b^	NA	NA
Greater California	1.17 (0.65-2.11)	.60
Hawaii^b^	NA	NA
Iowa^b^	NA	NA
Kentucky^b^	NA	NA
Los Angeles, California	1.63 (0.86-3.10)	.14
Louisiana	1.80 (1.06-3.07)	.03
New Mexico	2.85 (0.37-21.70)	.31
New Jersey	2.60 (1.53-4.40)	.001
Seattle (Puget Sound), Washington	1.87 (0.44-8.06)	.40
Utah^b^	NA	NA
Gleason grade groups 2-5		
Connecticut	1.36 (0.93-1.99)	.11
Detroit (Metropolitan), Michigan	1.65 (1.32-2.08)	<.001
Atlanta (Metropolitan), Georgia	1.88 (1.46-2.45)	<.001
Greater Georgia	1.29 (1.07-1.56)	.008
Rural Georgia	3.69 (0.96-14.20)	.06
San Francisco-Oakland metropolitan statistical area, California	1.25 (0.92-1.68)	.15
San Jose-Monterey, California	1.63 (0.88-3.01)	.12
Greater California	1.10 (0.91-1.36)	.32
Hawaii^b^	NA	NA
Iowa	0.72 (0.26-1.95)	.52
Kentucky	1.08 (0.75-1.57)	.68
Los Angeles, California	1.16 (0.93-1.46)	.17
Louisiana	1.28 (1.07-1.54)	.008
New Mexico	0.91 (0.29-2.84)	.88
New Jersey	1.52 (1.24-1.87)	<.001
Seattle (Puget Sound), Washington	1.42 (0.96-2.13)	.08
Utah	1.07 (0.15-7.85)	.95

Abbreviations: AHR, adjusted hazard ratio; NA, not applicable.

^a^ White men were used as the reference in each group.

^b^ Unable to calculate owing to insufficient events between races.

## Discussion

In this cohort study of the nationally representative SEER cancer registry, we found that statistically significant race-based differences in cancer-specific survival were confined mainly to low-grade prostate cancer and present in less than one-third of included SEER registries. These findings are consistent with the idea that racial differences in prostate cancer survival are subject to geographic variation.

Historically, racial differences in prostate cancer survival have been believed to be associated with a combination of differences in host genotype, differences in risk factors, and tumor biological differences, as well as differences in access to appropriate care.^11,12,13,14^ The relative contributions of these factors remain a topic of debate with substantial clinical and policy implications. For example, Jiang et al^15^ suggest that the differences are mostly associated with biological differences; therefore, they advocate for earlier and more rigorous prostate cancer screening in black men and early treatment of prostate cancer even at the low-risk stage.

Regarding low–Gleason grade group cancer, a 2018 study by Mahal et al^5^ reported that the greatest survival difference between black and white men was seen in patients presenting with low-risk prostate cancer. Our study adds to that evidence; after adjusting for patient, disease, and treatment characteristics, analyses demonstrated no significant racial difference in prostate cancer survival for most registries, regardless of disease grade at presentation.

Our analysis found that the differences in prostate cancer survival between black and white men for intermediate- and high-risk prostate cancer were present but lesser in magnitude compared with the difference in low-risk prostate cancer. These findings are in line with our 2019 study^3^ using data from the National Cancer Database, which found that when access to care, treatment, and cancer characteristics were accounted for, black race was not associated with a difference in overall survival for advanced prostate cancer. In fact, in that analysis, black race was associated with better overall survival.^3^ Data from several clinical trials point in the same direction. George et al^16^ reported that black men had better a progression-free survival rate compared with white men (16.6 months vs 11.5 months) when treated with abiraterone and prednisone. Moreover, a pooled analysis by Halabi et al^17^ of 9 randomized clinical trials for advanced prostate cancer found that overall survival was the same for black and white men. Taken together, these data may suggest that when black men are treated, they do equally well compared with white men. However, current evidence suggests that there is a significant gap in treatment rates between black and white men. For example, Underwood et al,^18^ as well as Moses et al,^19^ found that black and Hispanic men were significantly less likely to receive definitive therapy compared with white men. Surprisingly, higher tumor grade was associated with decreasing odds of definitive therapy for black and Hispanic men. In a 2018 study, Friedlander et al^2^ examined the facility-level variation in the use of definitive therapy among black and white men with intermediate- and high-risk prostate cancer and found that nearly half of the included institutions were more likely to provide definitive therapy to white men than black men. Similarly, a 2019 study^4^ found that men presenting with prostate cancer at hospitals that primarily treated people in racial/ethnic minority groups were less likely to receive definitive therapy and more likely to encounter delays to treatment, despite adjustment for race.

For low-risk prostate cancer, we found a statistically significant difference in prostate cancer–specific mortality between black and white men within 4 SEER registries: 2 from Georgia plus Louisiana and New Jersey. These findings provide clarification points to the 2018 study by Mahal et al^5^ that found worse outcomes in black men with low-risk prostate cancer. Many experts have interpreted those findings as a call for more aggressive treatment in black men presenting with low-risk disease. Our findings suggest that there may be specific demographic characteristic differences in prostate cancer care within specific areas of the US rather than a generalized association of worse survival for black men presenting with low-risk prostate cancer. Considering the complexity of low-risk prostate cancer management, for which the standard of care option is now active surveillance, requiring rigorous follow-up visits with repeated blood tests, physical examinations, biopsies, and optionally, imaging as well as genomic tests, gaps in access to quality care may exacerbate difference between demographic groups, whether stratified according to race, income, or education. For example, Krishna et al^20^ found that black men presenting with low-risk prostate cancer were followed less closely after an initial period of active surveillance than white men.

The findings of worse outcomes for black men in a wide spectrum of medical conditions specifically in the Atlanta area and in Georgia as a whole have been replicated in other non–prostate cancer–focused studies. Among large US cities, the widest gap in breast cancer mortality between black and white women was seen in Atlanta. These differences extend far beyond the scope of cancer care: the widest gap in US maternal mortality was seen in the state of Georgia.^21^ While our data cannot further characterize the causes of these differences, several hypotheses may be considered. Regardless of race, the mortality rates of cardiovascular disease, lower respiratory infections, meningitis, and asthma are also disproportionately high in Georgia.^22,23,24,25^ Translating these findings to prostate cancer, part of the reason may be associated with higher predisposition among inhabitants of Georgia. However, a combination of poor access to care cannot be discounted as a potential factor associated with racial differences for Georgia. In fact, a recent study showed that higher prostate cancer mortality incidence ratios in the state of Georgia were associated with worse health behavior and worse medical care.^26^ Our finding of a smaller magnitude but statistically significantly worse survival outcome for black men in New Jersey is also supported by prior literature.^27^ The cause for a stronger effect size seen in this registry is likely complex and multifactorial but may be associated with worse access to care among black men.

Although examining racial mortality differences according to registry cannot pinpoint causative or mediating factors or identify specific mechanisms accountable for these differences, our findings may help identify target areas for future research or interventions in both primary and secondary prevention. Specifically, if racial/ethnic differences in cancer outcomes are being driven by a small number of areas with large populations of racial/ethnic minority groups, then these represent important targets for addressing racial inequality. On the basis of previous research,^20,21,22,23,24,25^ such efforts should focus on identifying characteristics present in these areas, such as environmental risk factors and barriers to health care access. For example, Servadio et al^28^ showed that black individuals in the Atlanta area experience disproportionately high exposure to air pollution and lower access to green space compared with white individuals, both of which are associated with a higher prevalence of chronic obstructive pulmonary disease, chronic heart disease, and stroke. A similar study may help better understand the associations of such determinants with prostate cancer mortality. Regarding interventions, given such a large difference in mortality found in Georgia and New Jersey, a randomized clinical trial may help inform the value of systematic prostate cancer screening in at-risk populations, which remains an unresolved issue in the latest report from the US Preventive Services Task Force.^29^

### Limitations

Our study has several limitations that should be considered when interpreting our findings. This analysis is limited to the SEER registries, and it is plausible that other geographic areas of significant racial differences exist and would be worthy of further study. For example, there are several studies suggesting that similar racial differences in cancer mortality exist in South Carolina^30^; however, this possibility could not be examined, as it is not a SEER registry. Moreover, the significant disproportionate representation of white men in several registries poses a challenge, and for that reason, we adopted a methodological approach based on interaction term analysis rather than a separate registry-by-registry analysis. The SEER registries also lack data on patient comorbidities as well as quality of care processes (eg, caseload volume, provision of guideline-directed therapy), which can influence long-term mortality outcomes.

## Conclusions

After adjusting for patient, disease, and treatment characteristics, this cohort study found that population-level differences in prostate cancer survival among black and white men in the US were associated with a small set of geographic areas and with low-risk prostate cancer. While the cause of racial disparities in prostate cancer survival remains a topic of ongoing study, future studies and interventions should be targeted at settings where racial disparities are most pronounced.

## Notes

Supplement. **eTable.** Patient Characteristics by Registry
