# Supplementary material for: Geographic Distribution of Racial Differences in Prostate Cancer Mortality
Source: JAMA Netw Open. 2020 Mar 31;3(3):e201839. doi: 10.1001/jamanetworkopen.2020.1839 (PMC7109596; doi:10.1001/jamanetworkopen.2020.1839)
Supplement: Supplement. — eTable. Patient Characteristics by Registry [file jamanetwopen-3-e201839-s001.pdf]

Supplementary Online Content

Fletcher SA, Marchese M, Cole AP, et al. Geographic distribution of racial differences in prostate cancer mortality. *JAMA Netw Open*. 2020;3(3):e201839. doi:10.1001/jamanetworkopen.2020.1839

eTable. Patient Characteristics by Registry

This supplementary material has been provided by the authors to give readers additional information about their work.

© 2020 Fletcher SA et al. *JAMA Network Open.*

eTable. Patient Characteristics by Registry

AGE	<50	50-59	60-69	70-79	80+	Total
**Connecticut**	369	2,388	4,251	2,300	440	9,748
	4.46	4.3	4.3	4.16	3.72	4.24
**Cali other**	1,498	11,397	21,663	12,473	2,681	49,712
	18.12	20.51	21.91	22.58	22.68	21.64
**Atlanta**	558	2,615	3,968	1,777	332	9,250
	6.75	4.71	4.01	3.22	2.81	4.03
**Detroit**	554	3,195	5,171	2,955	766	12,641
	6.7	5.75	5.23	5.35	6.48	5.5
**Greater Georgia**	709	4,333	7,724	4,256	712	17,734
	8.58	7.8	7.81	7.71	6.02	7.72
**Hawaii**	70	550	1,166	793	282	2,861
	0.85	0.99	1.18	1.44	2.39	1.25
**Iowa**	191	1,981	3,670	2,116	554	8,512
	2.31	3.56	3.71	3.83	4.69	3.7
**Kentucky**	377	2,656	4,613	2,552	514	10,712
	4.56	4.78	4.67	4.62	4.35	4.66
**Los Angeles**	827	4,787	8,416	4,336	849	19,215
	10	8.61	8.51	7.85	7.18	8.36
**Louisiana**	565	3,815	6,665	3,796	893	15,734
	6.83	6.87	6.74	6.87	7.56	6.85
**New Jersey**	1,166	7,054	11,463	6,832	1,359	27,874
	14.1	12.69	11.59	12.37	11.5	12.13
**New Mexico**	107	991	2,003	1,206	239	4,546
	1.29	1.78	2.03	2.18	2.02	1.98
**Rural Georgia**	10	105	249	128	17	509
	0.12	0.19	0.25	0.23	0.14	0.22

© 2020 Fletcher SA et al. *JAMA Network Open.*

**SF-Oak**	448	3,221	5,953	3,149	755	13,526
	5.42	5.8	6.02	5.7	6.39	5.89
**San Jose-Mont**	232	1,773	3,195	1,731	476	7,407
	2.81	3.19	3.23	3.13	4.03	3.22
**Seattle-PS**	409	3,229	6,068	3,207	642	13,555
	4.95	5.81	6.14	5.81	5.43	5.9
**Utah**	177	1,481	2,642	1,627	308	6,235
	2.14	2.67	2.67	2.95	2.61	2.71
**Total**	8,267	55,571	98,880	55,234	11,819	229,771
	100	100	100	100	100	100
**Income**	**Low inc**	**High inc**	**Total**			
**Connecticut**	250	9,498	9,748			
	0.22	8.13	4.24			
**Cali other**	26,416	23,296	49,712			
	23.39	19.95	21.64			
**Atlanta**	667	8,583	9,250			
	0.59	7.35	4.03			
**Detroit**	6,298	6,343	12,641			
	5.58	5.43	5.5			
**Greater Georgia**	13,250	4,484	17,734			
	11.73	3.84	7.72			
**Hawaii**	401	2,460	2,861			
	0.36	2.11	1.25			
**Iowa**	7,065	1,447	8,512			
	6.26	1.24	3.71			
**Kentucky**	10,034	678	10,712			
	8.88	0.58	4.66			

© 2020 Fletcher SA et al. *JAMA Network Open.*

**Los Angeles**	19,215	0	19,215			
	17.01	0	8.36			
**Louisiana**	14,982	752	15,734			
	13.26	0.64	6.85			
**New Jersey**	5,283	22,583	27,866			
	4.68	19.34	12.13			
**New Mexico**	4,417	86	4,503			
	3.91	0.07	1.96			
**Rural Georgia**	509	0	509			
	0.45	0	0.22			
**SF-Oak**	0	13,526	13,526			
	0	11.58	5.89			
**San Jose-Mont**	0	7,407	7,407			
	0	6.34	3.22			
**Seattle-PS**	2,249	11,306	13,555			
	1.99	9.68	5.9			
**Utah**	1,911	4,324	6,235			
	1.69	3.7	2.71			
**Total**	112,947	116,773	229,720			
	100	100	100			
**Education**	**High edu**	**Low edu**	**Total**			
**Connecticut**	9,498	250	9,748			
	8.11	0.22	4.24			
**Cali other**	19,518	30,194	49,712			
	16.67	26.8	21.64			
**Atlanta**	8,583	667	9,250			

© 2020 Fletcher SA et al. *JAMA Network Open.*

	7.33	0.59	4.03			
**Detroit**	6,343	6,298	12,641			
	5.42	5.59	5.5			
**Greater Georgia**	3,374	14,360	17,734			
	2.88	12.75	7.72			
**Hawaii**	2,861	0	2,861			
	2.44	0	1.25			
**Iowa**	7,030	1,482	8,512			
	6	1.32	3.71			
**Kentucky**	1,072	9,640	10,712			
	0.92	8.56	4.66			
**Los Angeles**	0	19,215	19,215			
	0	17.06	8.36			
**Louisiana**	3,186	12,548	15,734			
	2.72	11.14	6.85			
**New Jersey**	16,630	11,236	27,866			
	14.2	9.97	12.13			
**New Mexico**	2,342	2,161	4,503			
	2	1.92	1.96			
**Rural Georgia**	0	509	509			
	0	0.45	0.22			
**SF-Oak**	11,251	2,275	13,526			
	9.61	2.02	5.89			
**San Jose-Mont**	6,064	1,343	7,407			
	5.18	1.19	3.22			
**Seattle-PS**	13,292	263	13,555			
	11.35	0.23	5.9			

© 2020 Fletcher SA et al. *JAMA Network Open.*

**Utah**	6,029	206	6,235			
	5.15	0.18	2.71			
**Total**	117,073	112,647	229,720			
	100	100	100			
**Insurance**	**Insured**	**Uninsured**	**Unknown**	**Total**		
**Connecticut**	9,178	271	299	9,748		
	4.36	8.16	1.87	4.24		
**Cali other**	47,866	439	1,407	49,712		
	22.74	13.22	8.8	21.64		
**Atlanta**	8,337	193	720	9,250		
	3.96	5.81	4.5	4.03		
**Detroit**	11,602	97	942	12,641		
	5.51	2.92	5.89	5.5		
**Greater Georgia**	15,921	339	1,474	17,734		
	7.57	10.21	9.22	7.72		
**Hawaii**	2,644	7	210	2,861		
	1.26	0.21	1.31	1.25		
**Iowa**	7,802	91	619	8,512		
	3.71	2.74	3.87	3.7		
**Kentucky**	10,062	166	484	10,712		
	4.78	5	3.03	4.66		
**Los Angeles**	18,342	536	337	19,215		
	8.72	16.14	2.11	8.36		
**Louisiana**	14,682	444	608	15,734		
	6.98	13.37	3.8	6.85		
**New Jersey**	21,843	445	5,586	27,874		
	10.38	13.4	34.92	12.13		

© 2020 Fletcher SA et al. *JAMA Network Open.*

**New Mexico**	3,786	62	698	4,546		
	1.8	1.87	4.36	1.98		
**Rural Georgia**	422	15	72	509		
	0.2	0.45	0.45	0.22		
**SF-Oak**	13,402	42	82	13,526		
	6.37	1.27	0.51	5.89		
**San Jose-Mont**	7,220	35	152	7,407		
	3.43	1.05	0.95	3.22		
**Seattle-PS**	12,189	90	1,276	13,555		
	5.79	2.71	7.98	5.9		
**Utah**	5,158	48	1,029	6,235		
	2.45	1.45	6.43	2.71		
**Total**	210,456	3,320	15,995	229,771		
	100	100	100	100		
**Gleason Grade Group**	**1**	**2**	**3**	**4**	**5**	**Total**
**Connecticut**	2,049	1,475	595	4,087	1,542	9,748
	3.91	4.03	4.3	4.26	4.98	4.24
**Cali other**	11,667	7,475	3,195	20,685	6,690	49,712
	22.24	20.44	23.11	21.56	21.59	21.64
**Atlanta**	2,256	1,540	596	3,544	1,314	9,250
	4.3	4.21	4.31	3.69	4.24	4.03
**Detroit**	2,294	2,230	762	5,260	2,095	12,641
	4.37	6.1	5.51	5.48	6.76	5.5
**Greater Georgia**	4,365	2,722	986	7,346	2,315	17,734
	8.32	7.44	7.13	7.66	7.47	7.72
**Hawaii**	548	477	233	1,100	503	2,861
	1.04	1.3	1.69	1.15	1.62	1.25

© 2020 Fletcher SA et al. *JAMA Network Open.*

**Iowa**	1,688	1,450	666	3,130	1,578	8,512
	3.22	3.97	4.82	3.26	5.09	3.7
**Kentucky**	2,446	1,707	557	4,577	1,425	10,712
	4.66	4.67	4.03	4.77	4.6	4.66
**Los Angeles**	4,442	3,294	1,116	8,000	2,363	19,215
	8.47	9.01	8.07	8.34	7.62	8.36
**Louisiana**	3,685	2,558	892	6,661	1,938	15,734
	7.03	6.99	6.45	6.94	6.25	6.85
**New Jersey**	6,641	4,295	1,430	12,298	3,210	27,874
	12.66	11.74	10.35	12.82	10.36	12.13
**New Mexico**	1,044	703	238	1,933	628	4,546
	1.99	1.92	1.72	2.01	2.03	1.98
**Rural Georgia**	131	72	27	193	86	509
	0.25	0.2	0.2	0.2	0.28	0.22
**SF-Oak**	3,120	2,082	784	5,791	1,749	13,526
	5.95	5.69	5.67	6.04	5.64	5.89
**San Jose-Mont**	1,603	1,107	430	3,381	886	7,407
	3.06	3.03	3.11	3.52	2.86	3.22
**Seattle-PS**	3,023	2,314	925	5,355	1,938	13,555
	5.76	6.33	6.69	5.58	6.25	5.9
**Utah**	1,450	1,068	391	2,593	733	6,235
	2.76	2.92	2.83	2.7	2.37	2.71
**Total**	52,452	36,569	13,823	95,934	30,993	229,771
	100	100	100	100	100	100
**Definitive Treatment**	**No Def Tx**	**Def Tx**	**Total**			
**Connecticut**	2,929	6,819	9,748			

© 2020 Fletcher SA et al. *JAMA Network Open.*

	4.14	4.29	4.24			
**Cali other**	16,219	33,493	49,712			
	22.94	21.05	21.64			
**Atlanta**	2,644	6,606	9,250			
	3.74	4.15	4.03			
**Detroit**	4,034	8,607	12,641			
	5.71	5.41	5.5			
**Greater Georgia**	4,755	12,979	17,734			
	6.73	8.16	7.72			
**Hawaii**	782	2,079	2,861			
	1.11	1.31	1.25			
**Iowa**	1,974	6,538	8,512			
	2.79	4.11	3.7			
**Kentucky**	2,814	7,898	10,712			
	3.98	4.96	4.66			
**Los Angeles**	6,299	12,916	19,215			
	8.91	8.12	8.36			
**Louisiana**	5,363	10,371	15,734			
	7.59	6.52	6.85			
**New Jersey**	6,407	21,467	27,874			
	9.06	13.49	12.13			
**New Mexico**	1,867	2,679	4,546			
	2.64	1.68	1.98			
**Rural Georgia**	135	374	509			
	0.19	0.24	0.22			
**SF-Oak**	5,518	8,008	13,526			
	7.81	5.03	5.89			

© 2020 Fletcher SA et al. *JAMA Network Open.*

**San Jose-Mont**	2,570	4,837	7,407			
	3.64	3.04	3.22			
**Seattle-PS**	4,612	8,943	13,555			
	6.52	5.62	5.9			
**Utah**	1,769	4,466	6,235			
	2.5	2.81	2.71			
**Total**	70,691	159,080	229,771			
	100	100	100			

© 2020 Fletcher SA et al. *JAMA Network Open.*
